# Discordance Between Cerebrospinal Fluid Biomarkers and Amyloid Positron Emission Tomography in Alzheimer’s Sheds Light on Clinical Trial Exclusion Criteria

**DOI:** 10.7759/cureus.13481

**Published:** 2021-02-22

**Authors:** Catherine K Major, Hamid R Okhravi

**Affiliations:** 1 Internal Medicine, Glennan Center for Geriatrics and Gerontology, Eastern Virginia Medical School, Norfolk, USA; 2 Internal Medicine: Geriatrics, Glennan Center for Geriatrics and Gerontology, Eastern Virginia Medical School, Norfolk, USA

**Keywords:** alzheimer's, discordance, dementia, biomarkers, clinical trials

## Abstract

Alzheimer’s disease (AD) is the most common type of dementia and affects millions of adults over the age of 60 years. Accurate diagnosis relies on multiple modalities, including neuropsychological testing, cerebrospinal fluid (CSF) biomarkers, and amyloid positron emission tomography (PET) imaging. Discordant results between CSF biomarkers and amyloid PET can be seen in up to 15% of cases and can lead to exclusion from clinical trials along with anxiety and confusion for the patient and family. We present two cases of discordance (CSF+, amyloid PET-) that resulted in the exclusion of both patients from two large anti-amyloid monoclonal antibody trials.

## Introduction

Alzheimer’s disease (AD) is the most common type of dementia and affects millions of adults over the age of 60 years. It is initially characterized by a slow progressive decline in declarative episodic memory. Impairment in executive function, as well as procedural memory, may not be apparent until later in the disease. The diagnosis and evaluation of AD involve interviewing and examining the patient, administrating a battery of neuropsychological tests, interviewing a proxy close to the patient, and using biomarkers. In a small subset of patients, Alzheimer’s disease can present with symptoms that overlap with primary tauopathies. Diseases such as frontotemporal dementia (Pick’s disease), progressive supranuclear palsy, and corticobasal degeneration fall into the category of primary tauopathies. In most cases, neuropsychological testing, a thorough history, and neuroimaging can aid in distinguishing between primary tauopathies and AD. Additionally, biomarkers in the cerebrospinal fluid (CSF) can illuminate the underlying pathology.

Biomarkers are used for the diagnosis and monitoring of the progression of those with AD. The main biomarkers currently used include structural magnetic resonance imaging (MRI), including volumetric MRI, fluorodeoxyglucose uptake on positron emission tomography (PET) (FDG-PET), PET amyloid imaging, and markers within the CSF. Structural MRI and volumetric studies can be used in the evaluation of patients with symptoms of AD. Patients with early AD often show decreased hippocampal volume and atrophy of the medial temporal lobe. Sequential monitoring of hippocampal volume loss over time with volumetric MRI can be used to predict the progression of AD [1]. FDG-PET can be used to detect decreased glucose metabolism in the brains of patients with mild cognitive impairment (MCI) and AD. A meta-analysis of the use of FDG-PET in the diagnosis of AD as compared to control has shown a sensitivity of 90% and specificity of 89% [2].

Amyloid PET can be used to assess the presence of beta-amyloid plaques and can affect clinicians’ diagnostic confidence and management plans significantly [3]. Studies have shown sensitivities between 89% and 97% and specificities between 63% and 93% with the use of amyloid-β PET in discriminating between AD and healthy controls [4]. CSF can be evaluated for low levels of amyloid-β (Aβ42), as well as increased total tau (T-tau), and phosphorylated tau (P-tau). Low levels of amyloid-β (Aβ42) can also be seen in non-AD dementias, such as Lewy body dementia, which highlights the importance of multiple reliable biomarkers to diagnose MCI and dementia due to AD.

Aβ42 levels in the CSF reflect the concentrations of soluble amyloid-β, which is hypothesized to correlate with amyloid-β deposits in the brain. A proposed model suggests that the earliest biomarkers of AD are decreased CSF Aβ42 and increased amyloid deposition in amyloid PET imaging, which can often be seen prior to the onset of symptoms [5]. There is also evidence that the magnitude of amyloid-β in the CSF plateaus as the patient approaches MCI and remains stable even as the disease progresses [5]. This is consistent with the idea that a reduction in CSF Aβ42 is an early marker of AD. Although the results between amyloid-β PET and CSF are usually concordant, discordant results are still seen up to 15% of the time (CSF+/PET- or CSF-/PET+), with a higher rate of discordance seen in subjective cognitive decline (15%) vs Alzheimer's dementia (5%) [6]. One possible hypothesis for this discordance is that soluble CSF Aβ42 decreases before above-threshold fibrillar amyloid-β deposits can be detected by PET imaging [7]. Patients with discordant Aβ markers are at increased risk of diagnostic progression than those who are concordant-negative [6]. This finding highlights the importance of continued monitoring in patients whose CSF and PET results are not concordant. The presence of one positive biomarker (CSF or PET) increases the risk of progression from subjective cognitive decline (SCD) to MCI or dementia, as well as progression from MCI to dementia [5]. A critical question regarding discordant cases is whether they are indicative of a positive AD status vs. an independent group with a unique clinical trajectory and characteristics. Discordance between the CSF and amyloid imaging can also have implications for clinical trial eligibility, as multiple anti-amyloid pharmaceutical trials require a positive amyloid imaging study. Discordant patterns may also delay therapeutic interventions or lead to the initiation of inappropriate medication secondary to an incorrect diagnosis. Additionally, this discordance can have a negative impact on the patient and their family if confusion arises regarding the diagnosis.

In 2018, the National Institute on Aging and Alzheimer’s Association (NIA-AA) published a research framework to biologically characterize AD by ATN biomarkers (Aβ deposition [A], pathologic tau [T], and neurodegeneration [N]) [8]. According to this system, each individual is classified for the presence of β-amyloid (CSF Aβ or amyloid PET: “A”), phosphorylated tau (CSF p-tau or tau PET: “T”), and neurodegeneration (atrophy on structural MRI, FDG PET, or CSF total tau: “N”). Due to this classification system containing options for both CSF biomarkers and imaging, questions can arise in cases of discordance [9].

We present two cases of discordance in amyloid-β PET and CSF biomarkers (PET-/CSF+). These patients were excluded from two major multi-center anti-amyloid monoclonal antibody clinical trials despite showing findings consistent with Alzheimer's on CSF. This case report describes cases of discrepancy given that many clinical trials exclude discordant patients with PET-/CSF+ biomarkers. Additionally, discussion of discordant results is crucial given that discordance could potentially represent up to 15% of those with AD [2,6].

## Case presentation

Case 1

**Figure 1:**
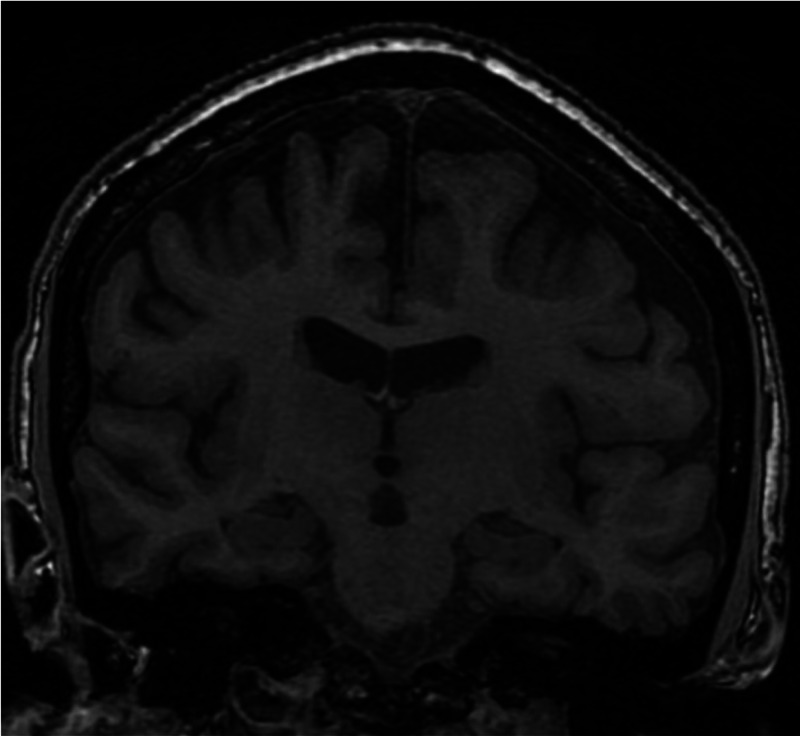
Coronal 3-D fast spoiled gradient echo (FSPGR) magnetic resonance (MR) image No significant findings are present.

A 74-year old male with a history of diabetes mellitus type two, hypertension, and hyperlipidemia presented to the Memory Clinic in September 2013 with a chief complaint of progressive short-term memory loss during the last three to four years. Functionally, he was independent in his activities of daily living with no changes in sleep or appetite. Vital signs and physical exam, including neurologic exam, were normal. His social history was significant for smoking one pack per day for many years and 16 years of education. Family medical history is significant for a maternal aunt diagnosed with Alzheimer’s dementia in her 70s. Initial assessment, including the administration of a 90-minute battery of neuropsychological testing, revealed mild impairments in list learning, delayed recall, and recognition pattern in verbal memory. Therefore, a diagnosis of single-domain amnestic mild cognitive impairment (aMCI) was made. Magnetic resonance imaging (MRI) of the brain was obtained, which showed no significant hippocampal volume loss by visual rating or significant white matter disease (Figure 1).

**Figure 2:**
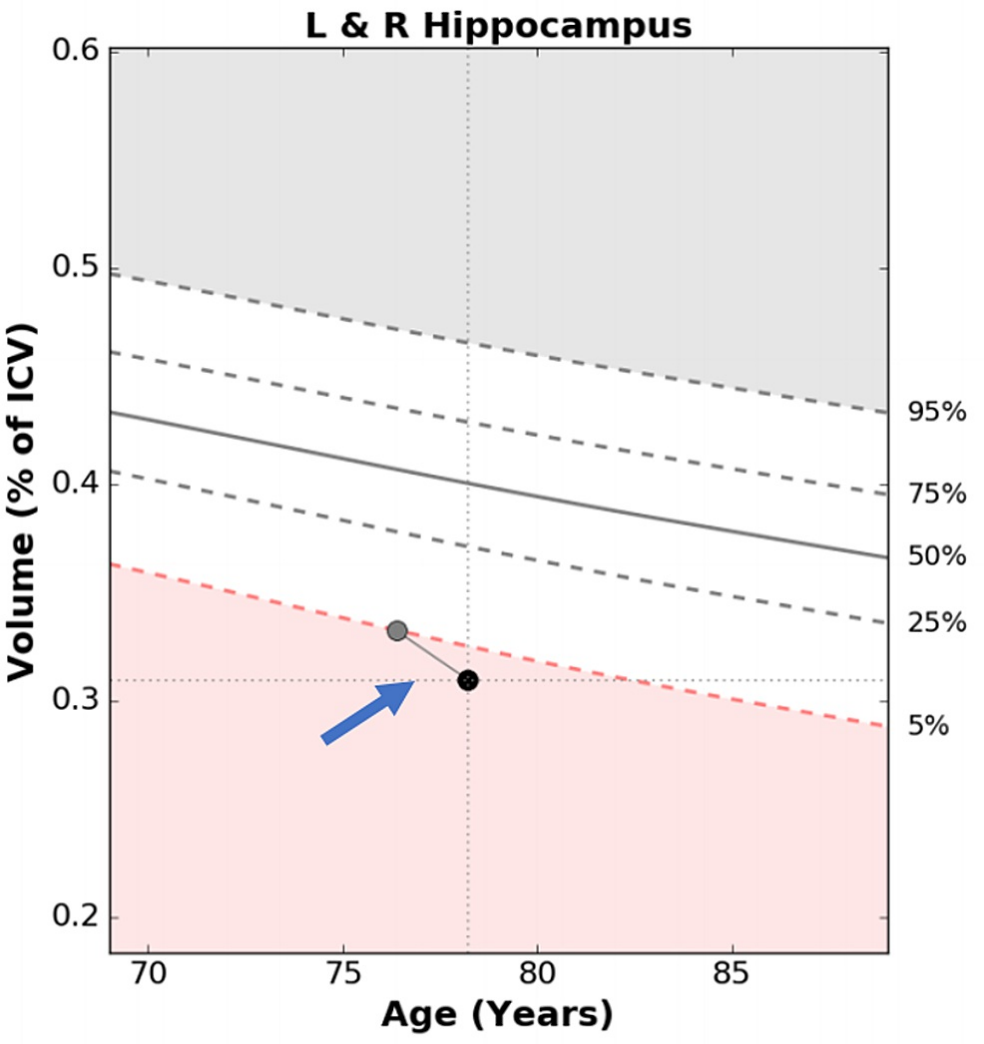
Volumetric magnetic resonance imaging (MRI) for Patient 1 in 2015 and 2016 The gray circle represents the patient's hippocampal volume in 2015 (5%) and the black circle represents the patient's hippocampal volume in 2016 (3%).

**Figure 3:**
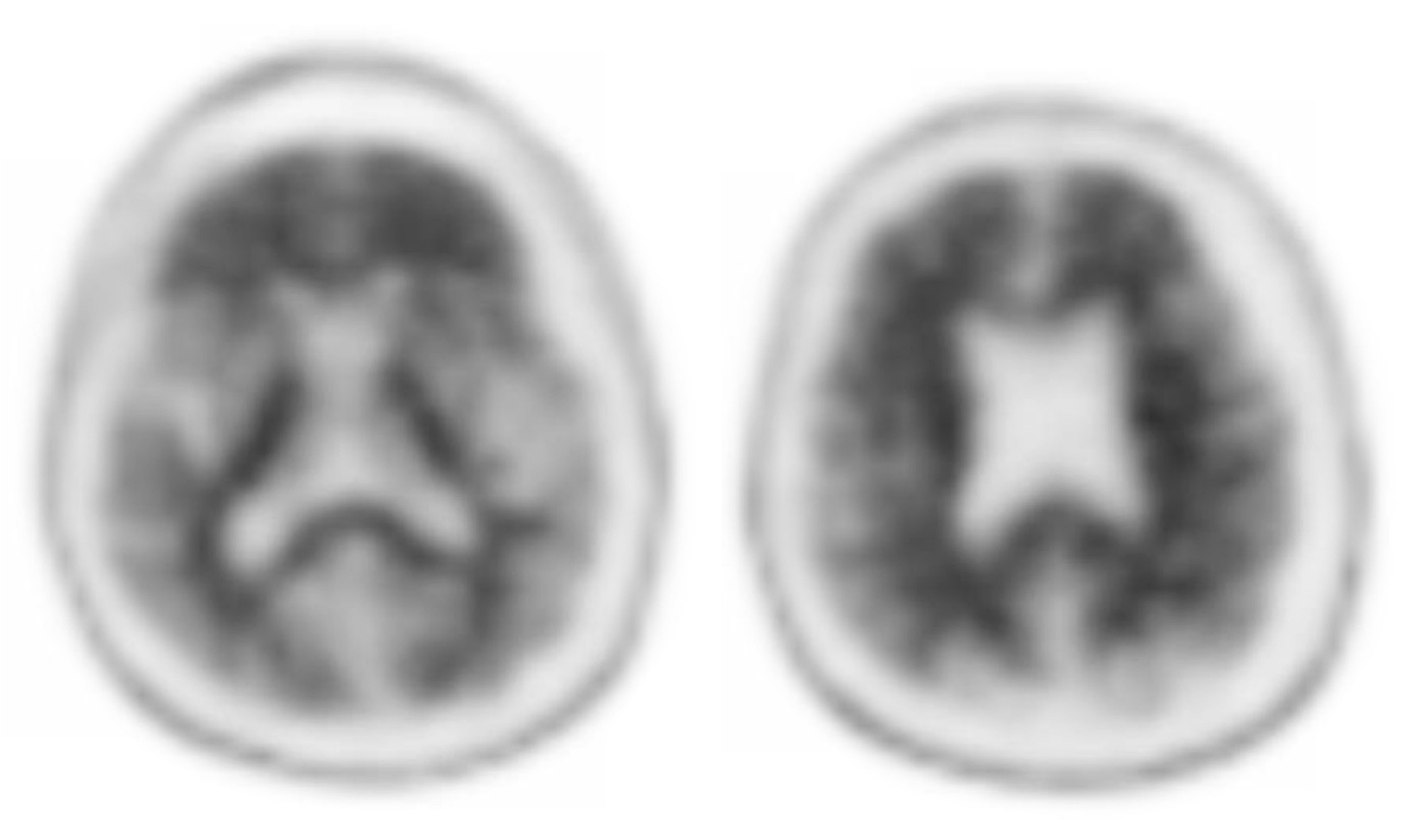
Amyloid imaging for Patient 1 that was read as negative

After a couple of years, the patient underwent a volumetric MRI, which revealed a hippocampal volume of 5% as compared to his age-and gender-matched peers (Figure 2). One year later, a second volumetric MRI demonstrated further hippocampal volume loss from 5% to 3% (Figure 2). He underwent amyloid imaging as part of an observational research study, which was read as positive for moderate to frequent neuritic plaques in the cortex. Considering his amnestic cognitive profile and hippocampal atrophy with accelerated hippocampal volume loss, a diagnosis of amnestic MCI due to AD with the highest probability was made. He was then referred for a major multi-center anti-amyloid antibody clinical trial. As part of the study requirement, he underwent another amyloid imaging. Despite the first reported positive amyloid imaging, the second one (part of the clinical trial) was read as negative (Figure 3). As a result, he did not qualify for participation in the clinical trial.

**Figure 4:**
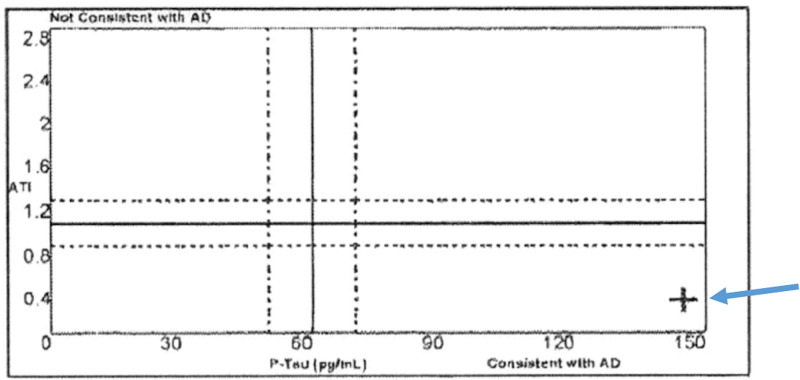
Cerebrospinal fluid (CSF) results for Patient 1 with phosphorylated tau (P-tau) and the Aβ42/total tau index (ATI), consistent with the diagnosis of Alzheimer's The blue arrows point toward the patient's value, which is represented by the black cross in the image.

Upon request, his initial amyloid imaging was also read by the central reading at the study site and that too was read as negative. This created a significant level of diagnostic uncertainty and increased anxiety in the patient and his family. To further clarify the accurate diagnosis, in October 2019, he underwent a lumbar puncture for CSF analysis for AD biomarkers. The CSF study revealed biomarkers indicative of Alzheimer’s disease (Figure 4). These results demonstrate a case where there is a discrepancy between the amyloid imaging study and CSF results. Classification within the A/T/N framework would be A+/T+/N+ or A-/T+/N+ depending on whether amyloid PET or CSF Aβ42 is utilized.

Case 2

**Figure 5:**
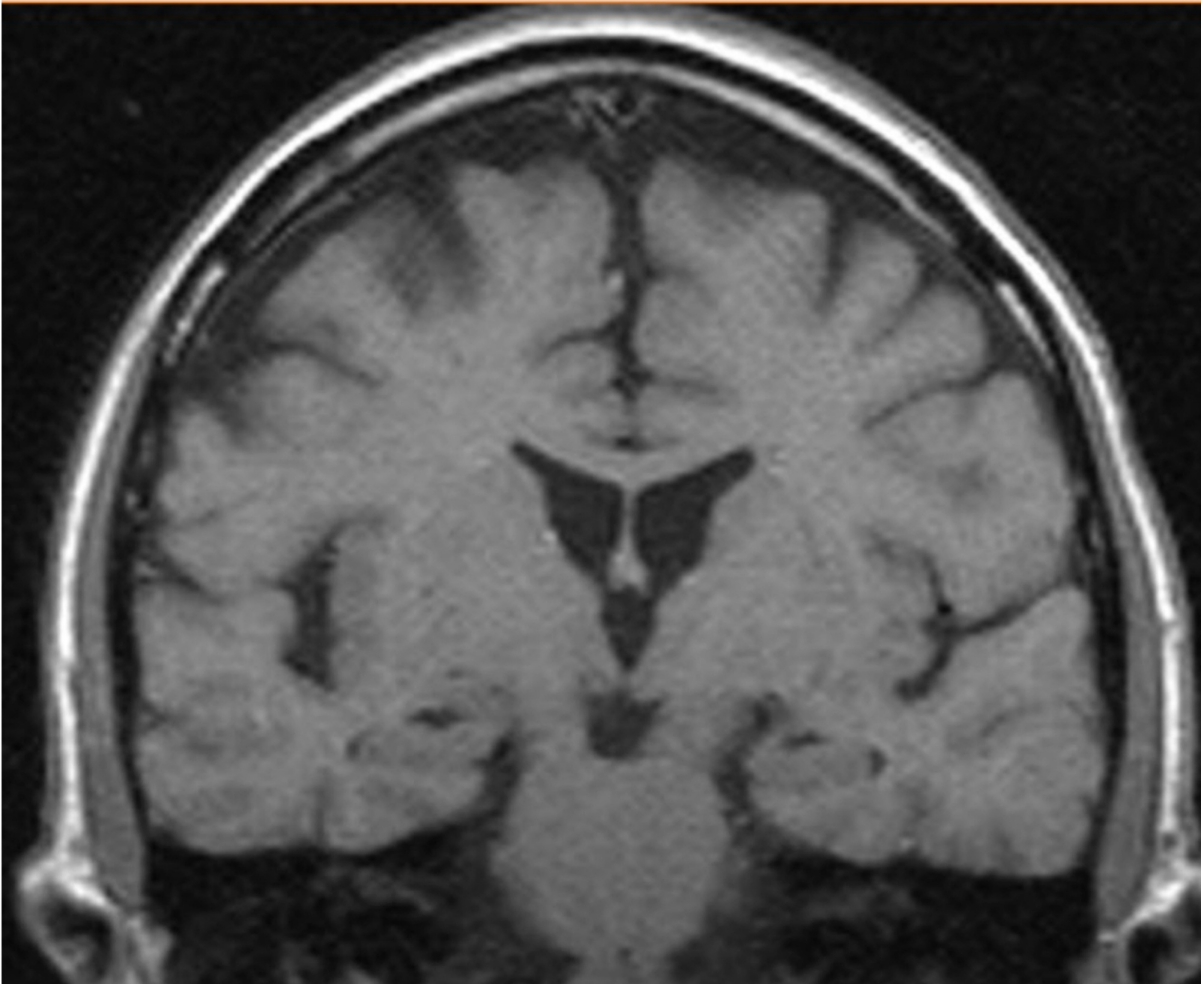
Coronal T1 weighted magnetic resonance imaging (MRI) scan of Patient 2, two years before his initial presentation No evidence of vascular pathology.

A 75-year old male with a history of depression presented to the Memory Clinic in March 2017 with the chief complaint of progressive short-term memory loss during the last three to four years. Functionally, he was independent in his activities of daily living. Vital signs were within normal limits and physical exam, including neurologic exam, was unremarkable. His social history was negative for tobacco or alcohol use and notable for a doctoral degree. Family medical history is significant for unspecified dementia in the patient’s mother. Initial assessment, including administration of a 90-minute battery of neuropsychological testing, revealed isolated impairment in verbal and visual memory. Therefore, a diagnosis of amnestic mild cognitive impairment, single domain was made. Magnetic resonance imaging from 2015 showed normal hippocampal volume by visual rating, with no evidence of vascular pathologies to explain his cognitive decline (Figure 5).

**Figure 6:**
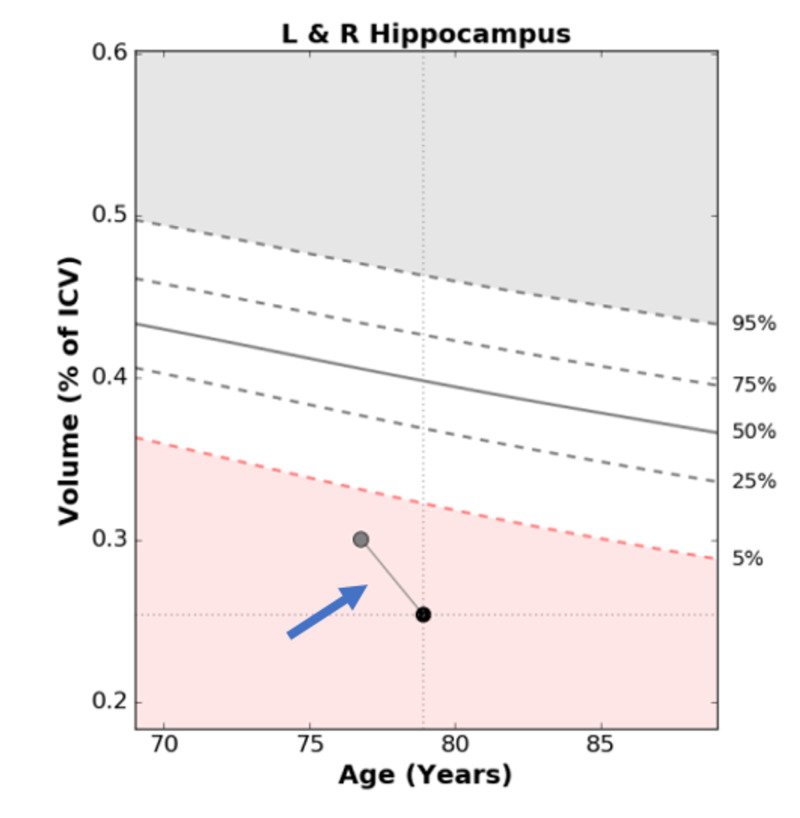
Volumetric magnetic resonance imaging (MRI) for Patient 2 in 2017 and 2020. The grey circle represents the patient's hippocampal volume in 2017 (3%) and the black circle represents the patient's hippocampal volume in 2020 (1%).

**Figure 7:**
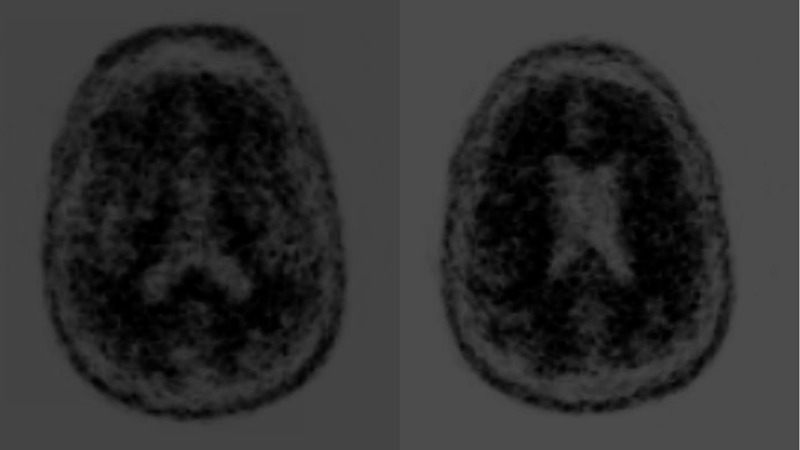
Amyloid imaging for Patient 2 that was read as negative.

**Figure 8:**
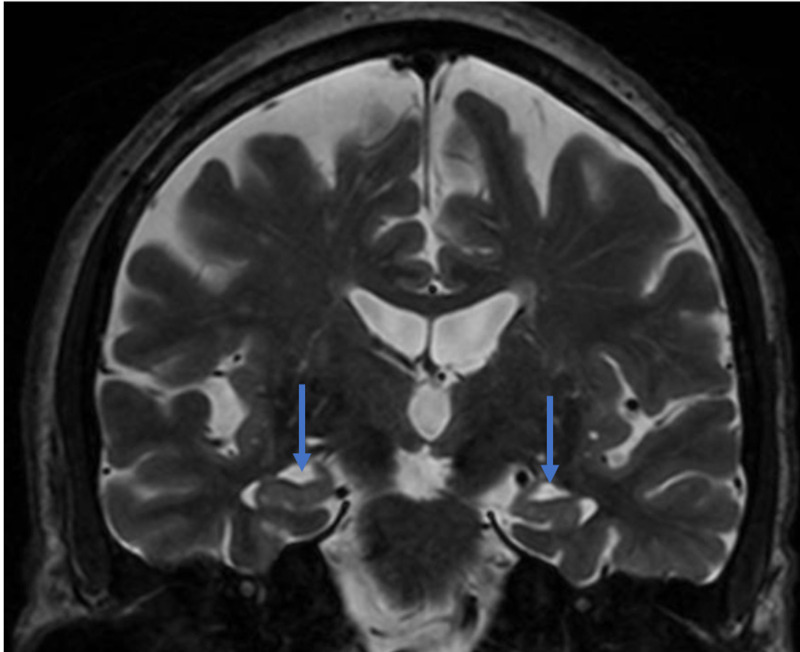
Coronal T2 weighted magnetic resonance imaging (MRI) scan of patient 2, in the stage of mild cognitive impairment. Coronal T2 sequences in patient 2 with blue arrows pointing towards volume loss in the hippocampus.

Volumetric MRI was performed in 2017, which demonstrated a hippocampal volume of 3% as compared to age-and gender-matched peers, consistent with hippocampal atrophy (Figure 6). Considering the amnestic profile associated with hippocampal atrophy, a diagnosis of probable Alzheimer’s disease was made, and he was referred to participate in an anti-amyloid study. He was excluded due to negative amyloid imaging (Figure 7). In August 2019, he underwent repeat neuropsychological testing, which was unchanged from 2017. In March 2020, he underwent MRI with volumetric studies, which showed accelerated hippocampal volume loss (Figure 6, Figure 8).

In August 2020, he underwent repeat neuropsychological testing, which demonstrated a further decline in his cognitive skills. In September 2020, he underwent a lumbar puncture which was consistent with a diagnosis of Alzheimer's (Table 1). He was referred to another anti-amyloid study and was excluded due to the history of negative amyloid imaging, despite his CSF positivity for the Alzheimer's biomarkers. Classification within the A/T/N framework would be A+/T+/N+ or A-/T+/N+ depending on whether amyloid PET or CSF Aβ42 is utilized.

**Table 1:** Cerebrospinal fluid (CSF) results for Patient 2 consistent with a diagnosis of Alzheimer's T-tau, total tau; P-tau, phosphorylated tau; ATI, Aβ42/total tau index; pg, picogram; ml, milliliter

Biomarker	Concentration (Reference)
Beta-amyloid 42	134.3 pg/ml (> 416 pg/ml)
T-tau	666.8 pg/ml (> 76.7 pg/ml)
P-tau	106.8 pg/ml (> 36.7 pg/ml)
ATI	0.13 pg/ml (< 1.0 pg/ml)

## Discussion

Discordance between amyloid PET and CSF could occur for a variety of reasons. Evidence has demonstrated that CSF may be abnormal years prior to abnormalities seen on imaging [10]. This highlights the importance of using CSF as a complementary marker for Alzheimer’s in clinical trials, given that many patients may have negative imaging findings while simultaneously having CSF abnormalities. Another possibility is that each abnormality represents a different aspect of the disease process. Amyloid PET positivity is more common as the disease progresses [11].

An important consideration with amyloid PET discrepancies is that amyloid PET imaging must be read by nuclear medicine specialists, and there could be variation in how those individuals interpret the scans. Amyloid PET imaging can be read visually or quantitatively through standardized uptake value ratios (SUVR). Discordance between visual and quantitative rating can be seen in up to 13% of cases, with quantitative rating categorizing more patients as positive on amyloid PET imaging [12]. The visual analysis appears to be more specific (96% vs 90%) than quantitative analysis but is less sensitive (79% vs 85%) [12]. The decreased sensitivity of visual analysis could lead to the exclusion of patients who have a lower burden of Aβ from clinical trials.

The experience of readers appears to be a factor in the interpretation of images, with a more experienced reader having a higher level of agreement between images [13]. Therefore, results may vary depending on whether the patient’s images are read by an expert. This, in turn, may affect the ability of patients to participate in clinical trials. Additionally, higher rates of agreement are also seen in patients whose images are read at the same center rather than spread across multiple centers, which could be attributed to different techniques, level of training, and the individuals responsible for reading the images [12]. Compared to visual and quantitative reading, CSF Aβ is more frequently positive [6,12]. This discordance could be due to the skill level among readers or due to the patient having a relatively low burden of Aβ. Additionally, it is hypothesized that CSF abnormalities may be present prior to a positive read on amyloid PET imaging [5].

In our first case, the same scan was read as positive by one nuclear medicine specialist and negative by the second specialist, working with the clinical trial. Reader concordance has been documented in previous literature with a 93.2% concordance rate for positive scans and a 100% concordance rate for negative scans [14]. This highlights the importance of a standardized system given the potential for variability among clinicians reading the scans. The use of training programs could be beneficial in reducing this variability, especially in instances of different levels of prior training [15]. False-negatives can also occur on amyloid PET scans, which are hypothesized to be due to differences in Aβ composition. The arctic APP early-onset familial AD presents with non-fibrillar varieties of Aβ that are not well-visualized by amyloid PET [16].

The implications of amyloid PET and CSF discordance on the patient and their family cannot be understated. This problem can present two-fold. Anxiety can be experienced by both the patient and caregivers surrounding the diagnosis. Many clinical trials have primary and secondary endpoints measuring CSF and amyloid PET. Excluding individuals from clinical trials due to CSF/amyloid PET discordance can have an undue negative effect on the well-being of the patient and their families. To the best of our knowledge, except for two case reports (both CSF+/PET-), there have been no head-to-head cross-sectional or longitudinal studies to compare in-vivo discordant cases with neuropathological findings [16-17]. Given that the discordance rate can be as high as 15%, it is imperative to investigate the clinical and biomarker course of those with discordant biomarkers and the implications of discordance related to participation in clinical trials. Again, CSF+/PET- amyloid-β status could be an indicator of early cases of amyloid pathology. Discordant test results could also impact the design of clinical trials with disease-modifying agents. The relatively high frequency of discordant cases (15%), calls for longitudinal observational biomarkers studies and clinical trials, designed specifically for this discordant cohort.

## Conclusions

As life expectancy continues to increase, the prevalence of Alzheimer’s will likely increase given that the majority of those diagnosed are over the age of 60. This increased prevalence will further emphasize the need for improved therapies and inclusive clinical trials. These two cases demonstrate that biomarker discordance can obscure an accurate diagnosis of Alzheimer’s disease with implications for clinical trial eligibility and options for treatment.

## References

[1] Jack CR, Petersen RC, Xu Y (2000). Rates of hippocampal atrophy correlate with change in clinical status in aging and AD. Neurology.

[2] Bloudek LM, Spackman DE, Blankenburg M, Sullivan SD (2011). Review and meta-analysis of biomarkers and diagnostic imaging in Alzheimer's disease. J Alzheimers Dis.

[3] Rabinovici GD, Gatsonis C, Apgar C (2019). Association of amyloid positron emission tomography with subsequent change in clinical management among medicare beneficiaries with mild cognitive impairment or dementia. JAMA.

[4] Morris E, Chalkidou A, Hammers A, Peacock J, Summers J, Keevil S (2016). Diagnostic accuracy of (18)F amyloid PET tracers for the diagnosis of Alzheimer's disease: a systematic review and meta-analysis. Eur J Nucl Med Mol Imaging.

[5] Jack CR Jr., Knopman DS, Jagust WJ (2010). Hypothetical model of dynamic biomarkers of the Alzheimer's pathological cascade. Lancet Neurol.

[6] de Wilde A, Reimand J, Teunissen CE (2019). Discordant amyloid-β PET and CSF biomarkers and its clinical consequences. Alzheimers Res Ther.

[7] Vlassenko AG, McCue L, Jasielec MS (2016). Imaging and cerebrospinal fluid biomarkers in early preclinical Alzheimer disease. Ann Neurol.

[8] Jack Jr CR, Bennett DA, Blennow K (2018). NIA-AA Research Framework: toward a biological definition of Alzheimer’s disease. Alzheimers Dement.

[9] Milà-Alomà M, Salvadó G, Shekari M (2021). Comparative analysis of different definitions of amyloid-β positivity to detect early downstream pathophysiological alternations in preclinical Alzheimer. J Prev Alzheimers Dis.

[10] Palmqvist S, Mattsson N, Hansson O (2016). Cerebrospinal fluid analysis detects cerebral amyloid-β accumulation earlier than positron emission tomography. Brain.

[11] Mattsson N, Insel PS, Donohue M (2015). Independent information from cerebrospinal fluid amyloid-β and florbetapir imaging in Alzheimer's disease. Brain.

[12] Schreiber S, Landau SM, Fero A, Schreiber F, Jagust WJ (2015). Comparison of visual and quantitative florbetapir F 18 positron emission tomography analysis in predicting mild cognitive impairment outcomes. JAMA Neurol.

[13] Zwan MD, Ossenkoppele R, Tolboom N (2014). Comparison of simplified parametric methods for visual interpretation of 11C-Pittsburgh compound-B PET images. J Nucl Med.

[14] Shea YF, Barker W, Greig-Gusto MT, Loewenstein DA, DeKosky ST, Duara R (2018). Utility of amyloid PET scans in the evaluation of patients presenting with diverse cognitive complaints. J Alzheimers Dis.

[15] Buckley CJ, Sherwin PF, Smith APL, Wolber J, Weick SM, Brooks DJ (2017). Validation of an electronic image reader training programme for interpretation of [18F]flutemetamol β-amyloid PET brain images. Nucl Med Commun.

[16] Scholl M, Wall A, Thordardottir S (2012). Low PiB PET retention in presence of pathologic CSF biomarkers in Arctic APP mutation carriers. Neurology.

[17] Cairns NJ, Ikonomovic MD, Benzinger T (2009). Absence of Pittsburgh compound B detection of cerebral amyloid beta in a patient with clinical, cognitive, and cerebrospinal fluid markers of Alzheimer disease. A case report. Arch Neurol.

